# Pregabalin attenuates long-lasting post-inflammatory nociplastic mechanical sensitization in mice

**DOI:** 10.1016/j.ynpai.2023.100131

**Published:** 2023-04-28

**Authors:** Manami Yajima, Yukari Takahashi, Yae K. Sugimura, Fusao Kato

**Affiliations:** a Center for Neuroscience of Pain and Department of Neuroscience, The Jikei University School of Medicine, Tokyo, Japan; b Department of Dental Anesthesiology, School of Dental Medicine, Tsurumi University, Yokohama, Japan

**Keywords:** Gabapentinoids, Inflammation-induced widespread pain, von Frey filament test, Central sensitization, Ectopic sensitization, Orofacial inflammatory pain

## Abstract

Nociplastic pain, the most recently proposed mechanistic descriptor of chronic pain, is the pain resulting from an altered nociceptive system and network without clear evidence of nociceptor activation, injury or disease in the somatosensory system. As the pain-associated symptoms in many patients suffering from undiagnosed pain would result from the nociplastic mechanisms, it is an urgent issue to develop pharmaceutical therapies that would mitigate the aberrant nociception in nociplastic pain. We have recently reported that a single injection of formalin to the upper lip shows sustained sensitization lasting for more than 12 days at the bilateral hindpaws, where there is no injury or neuropathy in rats. Using the equivalent model in mice, we show that pregabalin (PGB), a drug used for treating neuropathic pain, significantly attenuates this formalin-induced widespread sensitization at the bilateral hindpaws, even on the 6 day after the initial single orofacial injection of formalin. On the 10th day after formalin injection, the hindlimb sensitization before PGB injection was no more significant in mice receiving daily PGB injections, unlike those receiving daily vehicle injections. This result suggests that PGB would act on the central pain mechanisms that undergo nociplastic changes triggered by initial inflammation and mitigate widespread sensitization resulting from the established changes.

## Highlights

• Effect of pregabalin on ectopic sensitization after transient inflammation was tested.
• Pregabalin mitigated mechanical hindpaw sensitization in a nociplastic pain model.
• Pregabalin was effective even at day 10 after onset of nociplastic widespread pain.
• Therapeutic potential is revealed by this novel model of nociplastic pain.

## 1 Introduction

To tackle the immense problem of chronic pain, a prominent worldwide health concern, it is necessary to develop effective and appropriate approaches to mitigate the aversive phenotypes of chronic pain. To achieve this goal, it is necessary to understand the neural mechanisms underlying various chronic pain symptoms such as widespread sensitization. To date, three distinct types of mechanisms have been proposed to underlie chronic pain (Kosek et al., 2016). The first type is nociceptive pain, a type of pain caused by the ongoing activation of nociceptors. The second type is neuropathic pain, which results from aberrant activity of the neurons of the somatosensory system due to injury or disease in this system. The third type, the most recently proposed mechanistic descriptor of chronic pain, is nociplastic pain, which is pain resulting from an altered nociceptive function without demonstratable nociceptor activation and injury or disease in the somatosensory system.

Evidence from clinical and preclinical animal studies has revealed that gabapentinoids, such as pregabalin (PGB), gabapentin, and mirogabalin, are effective in mitigating neuropathic pain (Kremer et al., 2016). However, it has not been established whether gabapentinoids also improve the symptoms of nociplastic pain. This is primarily due to the absence or paucity of established preclinical models for nociplastic pain showing specifically sensitized nociception without nerve injury or inflammation, unlike nociceptive and neuropathic pain (Deuis et al., 2017).

We recently reported that an ectopic sensitization model produced by upper lip injection of formalin shows sustained sensitization lasting for more than 12 days in the bilateral hindpaws after a single injection of formalin to one side of the face (Sugimoto et al., 2021). Interestingly, this post-inflammatory sensitization is attenuated by a chemogenetics-mediated suppression of the excitability of central amygdala (CeA) neurons, indicating that the augmented activity of the CeA in this model plays an essential role in sensitization (Miyazawa et al., 2018, Sugimoto et al., 2021). As this model has no inflammation or injury at the sensitization site, we consider it suitable for testing drug effects on nociplastic pain with central (intra-brain) sensitization. Indeed, we recently reported that PGB significantly attenuated this bilateral sensitization in this model for approximately a few hours post-injection (Yajima et al., 2022). This result suggests that PGB might also act on the central pain network with nociplastic changes and mitigate widespread sensitization. However, in that report, the anti-sensitization effect of PGB was confirmed only at 3.5 h to 9 h after the initial inflammation (Fig. 4 in (Yajima et al., 2022)). If PGB mitigates widespread sensitization by affecting the central mechanism underlying its manifestation, its effect would be expected to last until the later stage of sustained sensitization in this model. To determine the mechanism of the anti-sensitization effect of PGB, we examined whether PGB could attenuate widespread sensitization at later stages after the initial single inflammatory event. Here we show that PGB significantly attenuates formalin-induced widespread sensitization in the bilateral hindpaws even 6 days after the initial single orofacial injection of formalin, and, on the 10th day after formalin injection, the hindlimb sensitization before PGB injection was no more significant in mice receiving daily PGB injections, unlike those receiving daily vehicle injections.

## 2 Materials and methods

The manipulation of the mice followed the Guidelines for the Proper Conduct of Animal Experiments of the Science Council of Japan (2006), and all experimental procedures were approved by the Institutional Animal Care and Use Committee of Jikei University (2022-048). We handled the mice according to our previous study using rats (Sugimoto et al., 2021, Yajima et al., 2022). Briefly, C57BL/6J mice (C57BL/6JJmsSlc; Japan SLC, Inc., Shizuoka, Japan) were housed in isolated ventilation cages at 5–6 mice/cage with free access to food and water and placed in a temperature/humidity-controlled room with a light/dark cycle (7:00–19:00, white light; 19:00–0:00, red light; 0:00–7:00 dark).

We created an orofacial inflammatory pain model by subcutaneous injection of 20 µL of 5% formalin solution (diluted from 37% solution, Nacalai Tesque Inc., Kyoto, Japan) into the left upper lip of the mice, just lateral to the nose, using a syringe with a 30-gauge needle (Becton, Dickinson and Company, Fukushima, Japan) under brief 5% isoflurane anesthesia. All animals injected with formalin displayed typical face-rubbing behavior lasting <60 min, a sign of acute nociceptive and inflammatory responses (Miyazawa et al., 2018), which was not analyzed in detail in this study. We estimated a 50%-threshold for the paw withdrawal responses (50% paw withdrawal threshold, PWT_50_) at the bilateral hindpaws using a calibrated series of von Frey filaments (0.008 – 2 g; North Coast Medical, Inc., Gilroy, CA, USA) and up-and-down methods (Chaplan et al., 1994). The von Frey test was performed in a mesh-floored chamber with a dim light on a two- or three-times a day basis (pre-drug injection, 5 h post-injection for PGB, pre-drug, 2 h and 5 h post-injection for celecoxib (Clx)), which was started on the following day post-formalin (Day 1) and repeated on days 3–4, 6–7, and 10 (Day 3–4, Day 6–7, and Day 10, respectively). The PWT_50_ values at the left and right hind paws were always measured in pairs and are shown as mean values indicated as “mean PWT_50_″ as sensitization always occurs bilaterally in this model (Sugimoto et al., 2021). We have already confirmed that there is no systemic difference between the PWT_50_ values from the right and left hindlimbs during the course of acute PGB treatment in the same model of rats (Yajima et al., 2022). After the von Frey test, the mouse was immediately returned to the home cage. PGB C-V (Sigma-Aldrich, Saint-Louis, MO, USA) was dissolved in 0.9% saline at 10 mg/mL and used for intraperitoneal injections (dose, 30 mg/kg body weight, i.e., 3 mL/kg). The same volume of 0.9% saline solution was administered and called ”vehicle” to PGB. Celecoxib (Selleck Biotech, Tokyo, Japan) was dissolved in 2% dimethyl sulfoxide (DMSO), 30% polyethylene glycol 300, 5% Tween-80, and 63% ddH_2_O at 2 mg/mL and used at a dose of 20 mg/kg body weight. DMSO solution without celecoxib was dissolved in the same solution and used for vehicle injections. Blood samples (0.7 mL) were collected transcardially into EDTA-2K coated tubes from mice 1, 3, 6, and 10 days after formalin or saline injection into the upper lip and from those without any prior injection (four mice for each condition) for the enzyme-linked immunosorbent assay (ELISA) for IL-1β (MLB00C; Quantikine, R&D) and TNF-α (MTA00B; Quantikine, R&D). After performing assays according to the manufacturer-recommended protocol, the optical density was evaluated using a microplate reader (Flex Station 3, Molecular Device, CA, USA). The optical density below the lower limit of the standard curve (12.5 pg/ml for IL-1β, 10.9 pg/ml for TNF-α) was judged “below the detection level.” Statistical comparisons were performed using SPSS 23 (IBM, Tokyo, Japan) and Igor Pro 9 (WaveMetrics, Lake Oswego, OR, USA) after converting original PWT_50_ values to logarithm values with the following approaches to test null hypotheses for each independent comparison: 1) unpaired t-tests between two groups (drugs versus vehicle) at each time point followed by post-hoc Benjamini-Hochberg false discovery rate (FDR) adjustment (Benjamini and Hochberg, 1995), 2) paired *t*-test for within-group comparisons (between pre-drug and post-injections of drugs) followed by post-hoc Benjamini-Hochberg FDR adjustment, 3) paired *t*-test for comparisons between the pre-drug value on the day 1 and that on the later days followed by post-hoc Benjamini-Hochberg FDR adjustment, and 4) paired *t*-test for comparisons between the pre-formalin value and each von Frey measurement followed by post-hoc Benjamini-Hochberg FDR adjustment. Details of the statistical tests are described in the figures and legends. The graphs, including the violin plot, were constructed with Igor Pro 9, using the implemented functions and procedures written by FK. Differences were considered significant at P < 0.05.

## 3 Results and discussion

**Fig. 1:**
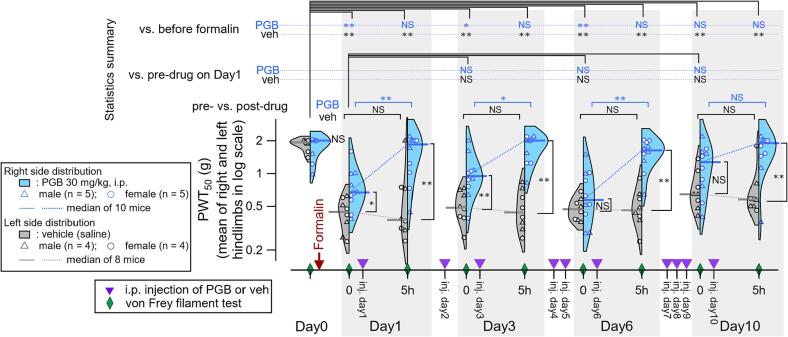
Effect of pregabalin (PGB, 30 mg/kg, i.p.) on the lowered mechanical threshold of the hindpaws in mice with latent inflammatory pain showing inflammation-induced widespread sensitization. Violin plots of 50%-paw withdrawal threshold (PWT_50_) of the hindpaws after upper lip injection of 5% formalin. Vertical axis, PWT_50_ values (mean of those at the right and left hindpaws). Horizontal axis, time points for the von Frey filament tests, the time after PGB injection (0 h and 5 h; violet reversed triangles), and the days after formalin injection (Day 0 - Day 10). Formalin was injected into the left upper lip of the mouse once, as indicated by the red arrow on day 0. PGB or its vehicle was administered intraperitoneally every day for ten days from the day after the formalin injection. On days 1, 3, 6, and 10 after formalin injection, von Frey filament tests were performed at 0 h (immediately before PGB or vehicle injection) and 5 h after PGB or vehicle injection (green rhombuses). The right (light-blue) and left (gray) sides of the violin plots for each von Frey measurement indicate the PGB- and vehicle-injected groups, respectively. Triangles and circles indicate the PWT_50_ of individual male (PGB, 5 mice; vehicle, 4 mice) and female (PGB, 5 mice; vehicle, 4 mice) mice, respectively. Horizontal bars in the left (vehicle) and right (PGB) of the violin plots are the median values for male and female mice in each group. The results and pairs for statistical comparisons across the time course are shown above the violin plot (multiple paired *t*-test with Benjamini-Hochberg false discovery rate post-hoc adjustment, *, p < 0.05; **, p < 0.01; NS, not significant). Signs to the right of each violin plot indicate the results of statistical comparisons between PGB- and vehicle-injected groups (unpaired *t*-test with Benjamini-Hochberg false discovery rate post-hoc adjustment, *, p < 0.05; **, p < 0.01; NS, not significant). Violin plots were made using the function implemented in Igor 9 with Scott bandwidth method and Gaussian Kernel. (For interpretation of the references to colour in this figure legend, the reader is referred to the web version of this article.)

We previously reported that a single injection of formalin into the upper lip results in ectopic sensitization, i.e., a long-lasting (up to 13 days) decrease in PWT_50_ in the bilateral hindpaws in rats (Sugimoto et al., 2021). Therefore, this is a good model for nociplastic pain, in which facial inflammation gives rise to mechanical sensitization at the distant hindpaw where there is no injury or inflammation. A single injection of formalin into the upper lip region of mice significantly lowered the PWT_50_ (measurement on Day 0 immediately before formalin injection and at time point 0 on Day 1 in Fig. 1), as observed in rats (Sugimoto et al., 2021). The PWT_50_ values on day 1, 3, 6, and 10 before PGB administration remained significantly smaller than the value on Day 0 in the vehicle group (measurements at “0h” in days 1, 3, 6, and 10 in Fig. 1). These results indicate that a single formalin injection into the upper lip caused sustained sensitization at the bilateral hindpaws in mice, in accordance with our previous observations in rats (Supplementary Fig. 2 in (Sugimoto et al., 2021)).

We then examined whether PGB mitigates the widespread sensitization at these later stages after the initial formalin injection. Fig. 1 shows the time course of the effect of PGB on Days 1, 3, 6, and 10 post-formalin (at “5h”). On these days, PGB was administered immediately after the first measurement of PWT_50_ of the day (“0”), and the von Frey filament test was repeated five hours after PGB injection (“5h”). PGB significantly elevated PWT_50_ 5 h post-administration, and this significant elevation was similarly observed until day 6. The post-PGB increase in PWT_50_ was not significant on Day 10 (Fig. 1), primarily due to the pre-PGB value at 0 h, which was slightly higher than that on days 1, 3, and 6 and no more significantly lower than that on Day 0 (the value before formalin) in the PGB group. In addition, PWT_50_ values 5 h after PGB injection did not differ significantly from the value on day 0, unlike that in vehicle-treated mice (Fig. 1). At 5 h post-PGB administration on days 1, 3, 6, and 10, the PWT_50_ consistently differed significantly between PGB and vehicle groups.

**Fig. 2:**
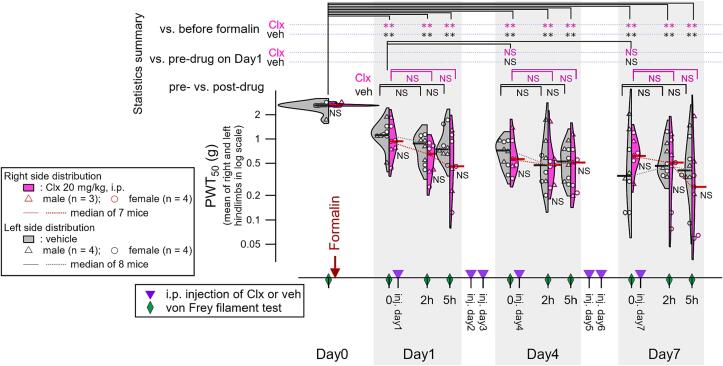
Effect of celecoxib (Clx, 20 mg/kg, i.p.) on the lowered mechanical threshold of the hindpaws in mice with formalin-induced widespread sensitization. Violin plots of 50%-paw withdrawal threshold (PWT_50_) of the hindpaws after upper lip injection of 5% formalin. The vertical axis shows the PWT_50_ values presented as the mean of the measurements at the right and left hindpaws. The horizontal axis shows the time points for the von Frey filament test (green rhombuses), time after Clx injection (violet reversed triangles), and days after formalin injection (days 0–7). Formalin was injected into the left upper lip of the mouse once, as indicated by the red arrow on day 0. Clx or its vehicle was administered intraperitoneally every day for seven days from the day after the formalin injection. At 1, 4, and 7 days after formalin injection, von Frey filament tests were performed at 0 (immediately before Clx/veh injection) and 2 and 5 h after Clx/veh injection (green rhombuses). The right (magenta) and left (gray) sides of the violin plots for each von Frey measurement indicate the Clx- and vehicle-injected groups, respectively. Triangles and circles indicate the PWT_50_ of individual male (Clx, 3 mice; vehicle, 4 mice) and female (Clx, 4 mice; vehicle, 4 mice) mice, respectively. Horizontal bars at the left (vehicle) and right (Clx) violin plots show median values for male and female mice in each group. The results and pairs for statistical comparisons across the time course are shown above the violin plot (multiple paired t-tests with Benjamini-Hochberg false discovery rate post-hoc adjustment, **, p < 0.01; NS, not significant). Signs to the right of each violin plot indicate the results of statistical comparisons between Clx- and vehicle-injected groups (unpaired *t*-test with Benjamini-Hochberg false discovery rate post-hoc adjustment, NS, not significant). Violin plots were made using the function implemented in Igor 9 with Scott bandwidth method and Gaussian Kernel. (For interpretation of the references to colour in this figure legend, the reader is referred to the web version of this article.)

To examine the possible involvement of inflammatory processes resulting from the post-injection consequences of upper lip formalin injection in sustained hindlimb sensitization, we examined the effect of a non-steroidal anti-inflammatory drug, celecoxib, on the formalin-induced hindlimb sensitization after its establishment. Celecoxib, at a dose reported to fully inhibit inflammatory pain behaviors after local formalin injection (Zhao et al., 2017), did not significantly affect the lowered PWT_50_ in mice with upper lip formalin injection at any time point from day 1 to day 7 post-formalin injection (Fig. 2). In addition, the plasma levels of IL-1β and TNF-α, major cytokines known to affect nociception, were below the detection limit of the kit used on days 1, 3, 6, and 10, despite the clear manifestation of hindpaw sensitization after upper lip formalin injection. These results suggest limited involvement of sustained systemic inflammation in the sustained manifestation of central sensitization.

The key finding of the present study is that PGB effectively reduces ectopic sensitization in the hindpaw initiated by a single upper lip injection of formalin, even on the sixth day after formalin injection. This acute effect of PGB was no more significant on Day 10, which was primarily due to the elevated pre-PGB PWT_50_ on this day in the mice receiving daily PGB on Days 1 – 9. In addition, the pre-PGB PWT_50_ on Day 10 was no more significantly lower than the pre-formalin value in this group, which was not the case in the group receiving daily vehicles on Days 1–9. We have already demonstrated that PGB administered 3.5 h after the upper lip formalin injection significantly mitigates the hindlimb sensitization up to ∼5 h after administration (Yajima et al., 2022). While this anti-sensitization effect might have involved the effects of PGB on early nociceptive and/or inflammatory events directly triggered by formalin injection (Hoffmann et al., 2022), the present results that PGB significantly attenuated sensitization at 1–6 days after a single formalin injection indicate that PGB exerts an anti-sensitization effect by affecting the established nociplastic changes that had occurred after the early and immediate post-formalin events (Miyazawa et al., 2018). Such post-formalin events might not involve early pro-inflammation properties of formalin as indicated recently (Hoffmann et al., 2022).

This effect of the daily single PGB injections was transient and faded within a day until post-formalin day 6, suggesting that PGB exerted only a transient inhibitory effect on the central network underlying this post-formalin sensitization. Although we found a tendency for sustained mitigation of central sensitization after repeated daily administration of PGB for 10 days, as evidenced by the absence of a significant decrease in the PWT_50_ compared to the pre-formalin value only in the PGB group (Fig. 1, statistic summary, top). However, the pre-PGB PWT_50_ on Day 10 after daily PGB injections for 9 days did not significantly differ from that measured after formalin injection on Day 1 either, indicating there was no significant recovery of the sensitization even after 9-days of daily PGB administration (Fig. 1, statistic summary, middle). It is speculated that the sensitization on Day 10 might reflect the mixed influences of the spontaneous recovery, repeated PGB effects and persistent nociplastic pain. Additional experiments with a longer period of repeated administration as well as an intervention to prolong the duration of stable nociplastic sensitization, such as that shown in a recent paper using post-injury thermal stimulation (Hankerd et al., 2022), would be necessary.

This would be an important subject of future studies, as PGB is used in clinical setups of chronic pain with a longer period of drug administration. In addition, it is worth noting that the present findings do not necessarily indicate that PGB would be effective in all types of clinical chronic pain with nociplastic pain. Indeed, PGB is highly effective in improving fibromyalgia but only in one out of 10 patients (Derry et al., 2016). An interesting aspect of the present model is that there were no apparent systemic sex-dependent differences (triangle and circle markers in Fig. 1, Fig. 2) in contrast to a marked sex-dependent difference in another nociplastic pain model of mice (Hankerd et al., 2022). This point is interesting to understand the female-dominant nature of fibromyalgia. It is imperative to develop various types of preclinical models representing various etiologies of clinical nociplastic pain and also to re-evaluate the possible involvement of nociplastic factors in the conventional models of chronic pain to develop novel treatments for distinct classes of nociplastic pain.

Tanabe et al. reported that the analgesic effect of PGB was entirely dependent on the injury associated with the tested site (i.e., hindpaw in their sciatic nerve section model), regardless of the site of administration (i.e., the brain or spinal cord) (Tanabe et al., 2008). In addition, it has been reported that the expression upregulation of α_2_δ subunits of the voltage-dependent Ca^2+^ channels, the most plausible target molecule of PGB (Patel and Dickenson, 2016), at the spinal dorsal horn by spinal nerve neuropathy is a requisite for the anti-sensitization effect of PGB in the neuropathic pain model (Kusuyama et al., 2018, Patel and Dickenson, 2016). In these contexts, the present finding that PGB attenuated sensitization at the bilateral hindlimbs in the absence of injury or neuropathy of the innervating nerves is exceptional.

A most plausible interpretation of this exceptional effect is that PGB affected neuronal signaling in the central network that controls tactile mechanical sensitivity and withdrawl responses involving neurons expressing α_2_δ subunits in this model. The in situ hybridization data indicate that they are not only expressed in the spinal dorsal horn and spinal nucleus of the trigeminal nerve but also rich in the central structures involved in pain signaling, such as the parabrachial nucleus, basolateral amygdala (BLA), CeA, and ventrolateral periaqueductal grey (Allen Mouse Brain Atlas). Indeed, we have demonstrated that PGB is highly effective in reducing excitatory synaptic transmission between the BLA and CeA only in brain slices from mice with an intraplantar injection of formalin 8 h before brain removal (Yamamoto et al., 2021). In addition, local injection of calcitonin gene-related peptide into the right CeA is sufficient to mitigate the sensitization at the bilateral hindpaw in the upper lip formalin model of the rats (Sugimoto et al., 2021), suggesting a primary role of the CeA and associated network in this formalin-induced ectopic sensitization and its maintenance. Therefore, it is plausible that the attenuation of widespread sensitization by PGB results from its effect on synaptic transmission between the central structures regulating nociceptive sensitivity (Sadler et al., 2017, Sugimoto et al., 2021, Wilson et al., 2019).

Other possibilities that might account for the mitigating effect of PGB on the remote and ectopic sensitization in the hindlimb might include 1) direct modulation of the spinal cord network underlying the paw withdrawal by PGB (Hendrich et al., 2012) and 2) reduced influence of systemic inflammation rising from lip injury by formalin solution on the sensitization by PGB. The former possibility could not be fully ruled out, and could be directly examined by injecting PGB into the spinal cord. However, as it has been shown that the spinal effect of PGB depends on spinal nerve injury (Kusuyama et al., 2018, Tanabe et al., 2008), which is not present in the present model, and that PGB does not affect motor function in non-injured mice (Tanabe et al., 2008), the information obtained by this interventional approach is limited. Although we did not evaluate the spinal expression of α_2_δ subunits in the present model, it is difficult to imagine that highly regional and limited inflammation at the face upregulates α_2_δ subunit expression in the dorsal horn without any increase in spinal nociceptive signals in this model. Possibility 2 is also less likely, as the level of serum cytokines was not beyond the detectable level at any stage, despite the manifestation of ectopic sensitization, which was resistant to NSAID treatment.

## Conclusion

Here, we provide evidence indicating that sensitization in regions distant from the site of initial transient inflammation is markedly attenuated by PGB even 6 days after the initial inflammation. This significant sensitization in the hindlimb on Days 1–6 after a single initial injection of formalin became non-significant on Day 10 in the mice receiving daily PGB. These results strongly support the notion that nociplastic sensitization at a site without injury, inflammation, and neuropathy is established through plastic changes in the central pain network that are consolidated to influence peripheral nociception persistently in widespread regions. As some of these brain networks express α_2_δ proteins, it is possible that PGB would affect these α_2_δ proteins and attenuate central sensitization. As such, nociplastic changes in the central pain network could be initiated by local and transient inflammation (Miyazawa et al., 2018) as well as by nerve injury (Ikeda et al., 2007, Nakao et al., 2012). It is also possible that the analgesic effects of PGB reported to date in preclinical models of neuropathic or inflammatory pain would also involve its effect on the nociplastic changes in the central pain network caused by neuropathy and inflammation.

## Funding

Japan Agency for Medical Research and Development (22ek0610026h0002).

## CRediT authorship contribution statement

**Manami Yajima:** Methodology, Conceptualization, Data curation, Formal analysis, Writing - original draft. **Yukari Takahashi:** Conceptualization, Data curation, Investigation, Methodology, Writing - original draft. **Fusao Kato:** Conceptualization, Visualization, Supervision, Funding acquisition, Formal analysis, Methodology, Project administration, Software, Writing - original draft, Writing - review & editing.

## Declaration of Competing Interest

The authors declare the following financial interests/personal relationships which may be considered as potential competing interests: Fusao Kato is a recipient of a collaborative study on the effects of novel gabapentinoids with Daiichi-Sankyo Co. Ltd.

## Data Availability

Data will be made available on request.
